# A novel nerve block and anatomy workshop for emergency medicine residents: A pilot study

**DOI:** 10.1002/ase.70240

**Published:** 2026-04-14

**Authors:** Geoffery D. Fernquist, Derek Harmon, Lauren D. Branditz, Andrew Kendle, Melissa M. Quinn

**Affiliations:** ^1^ Section of Anatomy, Department of Surgery University of Texas Southwestern Medical Center Dallas Texas USA; ^2^ Division of Anatomy, Department of Biomedical Education and Anatomy The Ohio State University College of Medicine Columbus Ohio USA; ^3^ Department of Emergency Medicine The Ohio State University Columbus Ohio USA; ^4^ Emergency Department University of California, San Francisco San Francisco California USA

**Keywords:** anatomy education, emergency medicine education, emergency medicine residency education, graduate medical education, nerve blocks, regional anesthesia, ultrasound‐guided regional anesthesia

## Abstract

Construct a workshop for emergency medicine (EM) residents to learn ultrasound‐guided regional anesthesia (UGRA) procedures and build confidence in performing those procedures. Use pre‐ and post‐workshop knowledge and confidence surveys to determine workshop effectiveness. We created a workshop using prosected donors, ultrasound (US) scanning on standardized patients, and needling technique on a phantom model to increase residents' knowledge and confidence for eight UGRA procedures. Pre‐ and post‐workshop assessments are used to measure confidence and knowledge gains. Forty‐four EM residents participated in the workshop. Residents scored significantly higher on the post‐workshop (*n* = 29) knowledge assessment compared to the pre‐workshop (*n* = 39) knowledge assessment (12.0 ± 2.0 vs. 8.44 ± 2.45; *p* < 0.001). Confidence increased significantly on the post‐workshop (*n* = 40) assessment compared to the pre‐workshop (*n* = 28) assessment (*p* < 0.001). “Extremely confident” and “very confident” statements increased from 11.1% to 45.1% of responses. The self‐efficacy gained by residents in this workshop can lead to increased patient safety as opioids are avoided in the emergency department. Workshops such as the one described in this manuscript provide residents and attendings opportunities to learn skills and knowledge for UGRA procedures. This workshop provides EM faculty with an effective environment in which to teach their residents proper skills for UGRA and can be easily replicated at other institutions. Future workshops should include assessment of clinical competency following training.

## INTRODUCTION

Pain is one of the most common reasons for patients to seek care in an emergency department (ED). As such, emergency medicine (EM) physicians must strive to become experts in providing pain management while focusing on safer alternatives to opioids.^1^, ^2^ The ongoing opioid crisis led the American College of Emergency Physicians (ACEP) to include ultrasound‐guided regional anesthesia (UGRA) as a “core skill” which is “not only within the scope of practice of emergency physicians, but which represents a core component of a multimodal pathway to control pain for patients in the ED”.^3^ The use of UGRA for pain management in patients with acute traumatic injuries as an opioid sparing best practice strategy is also supported by the American College of Surgeons.^4^

UGRA offers many benefits over opioids including shorter hospital stays, decreased complications, and improved pain control.^4^, ^5^ These measures lead to greater patient safety and improve long‐term functional outcomes.^6^ Additionally, ultrasound (US) use during nerve blocks improves efficiency and patient safety^3^ when compared to the blind approach.

While UGRA is increasingly becoming more popular within EM, there are institutions that either do not perform these procedures or perform few due to a lack of educational opportunities for residents.^5^ A survey by Amini et al.^5^ revealed that 103 EM residency programs (84% of respondents) perform UGRA, but most complete <50 total blocks per month. Tucker et al.^6^ reported that only 53% of EM residencies teach UGRA in their core curricula. Potential UGRA curricula for EM have been proposed,^6^, ^7^, ^8^ although none include outcomes assessments such as testing for anatomical knowledge and confidence in abilities to perform UGRA procedures.

Current UGRA curricular practices are extremely variable among programs,^5^ making it difficult to create a uniform curriculum among all EM residencies.^9^ One of the largest obstacles preventing programs from instituting an UGRA curriculum is lack of faculty confidence and training.^9^ However, previous studies have revealed how nerve block procedures can be learned with little time investment.^5^ With dedicated training, EM residents have shown competency in many nerve blocks including the femoral nerve^10^ and the interscalene^11^ blocks.

UGRA success relies on a thorough understanding of anatomical relationships, including the ability to interpret US imaging.^12^ Many residents have not formally reviewed anatomy since medical school,^13^, ^14^ suggesting a need for review.

Simulation and body donor labs are common training tools for UGRA education in other specialties. In one study, an anesthesiology residency program implemented a workshop where residents simultaneously reviewed anatomy on body donor specimens with UGRA instruction.^13^ A similar study taught anesthesiology residents using didactics followed by an anatomy review on body donors and US scanning instruction on standardized patients.^12^ A four‐week intervention for anesthesiology residents led to greater efficacy when performing the interscalene block under US guidance.^15^ Kim and Tsui^14^ found that anesthesiology residents who completed a 1‐h simulation training found greater success while performing UGRA procedures compared to others who did not complete such training.

Previous studies have coupled anatomy review on body donors with US scanning on models, but no other study has published an UGRA workshop for EM residents that combines anatomy review on body donors, dedicated US scanning practice on standardized patients, and in‐plane needling technique on phantoms.

The objective of this study was to pilot and evaluate a novel anatomical and US‐based workshop focusing on eight common nerve block procedures for EM residents. In this study, we used prosected body donors that highlight essential anatomical landmarks for these procedures in conjunction with US scanning on standardized patients and in‐plane needling technique on a phantom model.

## METHODS

### Workshop introduction

A workshop to improve resident anatomical knowledge and confidence for specific UGRA procedures was created using an anatomical review on prosected donors coupled with US imaging, US scanning practice on standardized patients, and in‐plane needle‐to‐target practice under direct ultrasound guidance. The anatomy review and UGRA workshop pilot study was approved by The Ohio State University Institutional Review Board (#2023E1285). This workshop was created using criteria from Curriculum Development for Medical Education.^16^ EM residency leadership at The Ohio State University (OSU) approved their residents to participate in a workshop focused on teaching UGRA procedures. Forty‐four EM residents participated in the workshop.

High‐yield UGRA procedures appropriate for this workshop were determined based on the expert consensus opinion of EM faculty members who are also members of the department's Division of Ultrasound. UGRA procedures included the: superficial cervical plexus block, serratus anterior plane block, ulnar nerve block, median nerve block, radial nerve block, erector spinae plane block, pericapsular nerve group (PENG) block, and tibial nerve block (at the ankle). Many of these procedures were indicated as essential blocks in a recent Delphi study.^8^

### Pre‐workshop assessments

The workshop began by administering two assessments. First, a pre‐workshop knowledge assessment, consisting of 16 multiple choice questions to test residents' anatomical knowledge of the previously mentioned eight UGRA procedures. Second, a 16‐question pre‐workshop confidence assessment asked residents to rate their confidence in recognizing anatomical landmarks for the same UGRA procedures using a 1 (Not at all Confident) to 5 (Extremely Confident) Likert scale. The pre‐workshop confidence assessment also asked participants to provide their name or answer security questions to link the assessments, state their post‐graduate year (PGY) with respect to time since medical school graduation, provide details about their most recent anatomy course, and how many nerve blocks they have either completed or observed thus far in their training.

Following the pre‐workshop assessments, EM residents were evenly divided into two groups for the workshop. The first group participated in an anatomy review using prosected donors under the direction of anatomy PhD students. The second group participated in US scanning on live models and in‐plane needling technique practice on phantom models under the direction of EM faculty members.

### Anatomy prosection review

The anatomy review required 50 min and used three prosected donors showing anatomy relevant for the UGRA procedures. Each prosected donor station included 1 anatomy graduate student instructing 3 to 5 participants. Anatomy review focused on nerves and the surrounding anatomical landmarks with respect to each UGRA procedure. These prosections helped the residents visualize the anatomy of these procedures and how to properly approach the targeted area with a needle. (A probe was used to demonstrate the needle position for each procedure.) In addition, images were used during the anatomy review to help the EM residents visualize the cutaneous distribution of the nerves. The images were found using various online resources, such as the NYSORA website.

On each of the three donors, the following areas were prosected: the superficial cervical plexus, lateral cutaneous branches of intercostal nerves within the plane between the serratus anterior and latissimus dorsi muscles, the radial nerve in the forearm/hand, the median nerve in the forearm/hand, the ulnar nerve in the forearm/hand, dorsal rami within the erector spinae muscle group (including visualization of transverse processes), the hip joint with the femoral and obturator nerves, and the tibial nerve within the tarsal tunnel.

### US instruction

Participants spent 50 min practicing their US scanning and in‐plane needling technique. First, sonographic anatomy relevant to each UGRA procedure was demonstrated by an EM attending physician or US fellow who was a part of the Department of Emergency Medicine Division of Ultrasound. Scanning was performed on Trained Simulated Ultrasound Patients (“TSUPs”) using Sonosite US machines. TSUPs are medical students who are trained to act as ultrasound models for purposes of instructional sessions.^17^

Following large group demonstrations by faculty members, learners were divided into six small groups. Groups were taken through each UGRA procedure by a Division of Ultrasound faculty member or fellow. These small groups included review of sonographic anatomy by the faculty member or fellow while performing a live scan demonstration on a TSUP. Discussions at each station included review of anatomy, including anatomic landmarks that should be identified, important structures to recognize and avoid, appropriate patient positioning, appropriate type and volume of anesthetic to be used for that specific procedure, risks relevant to all UGRA procedures, and risks specific to each block. Following demonstrations, each learner practiced hands‐on scanning skills including probe selection, transducer manipulation, and landmark identification by scanning on TSUPs under the supervision and guidance of Division of Ultrasound faculty and fellows.

### Needling technique instruction

**FIGURE 1:**
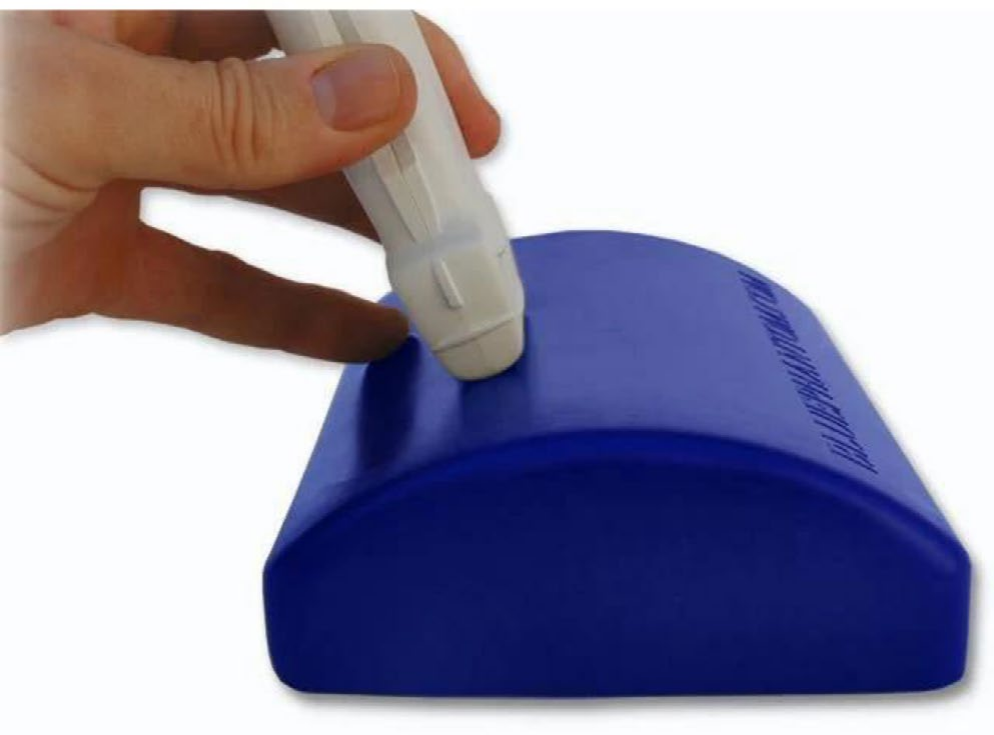
The regional anesthesia ultrasound training block.

At a separate station, learners performed in‐plane needle‐to‐target practice under direct ultrasound guidance. Two types of gel phantoms were used for this activity. The first was a regional anesthesia nerve block blue gel phantom (Figure 1). This device can be viewed and purchased on the website page, www.gtsimulators.com (Regional Anesthesia Ultrasound Training Block).

**FIGURE 2:**
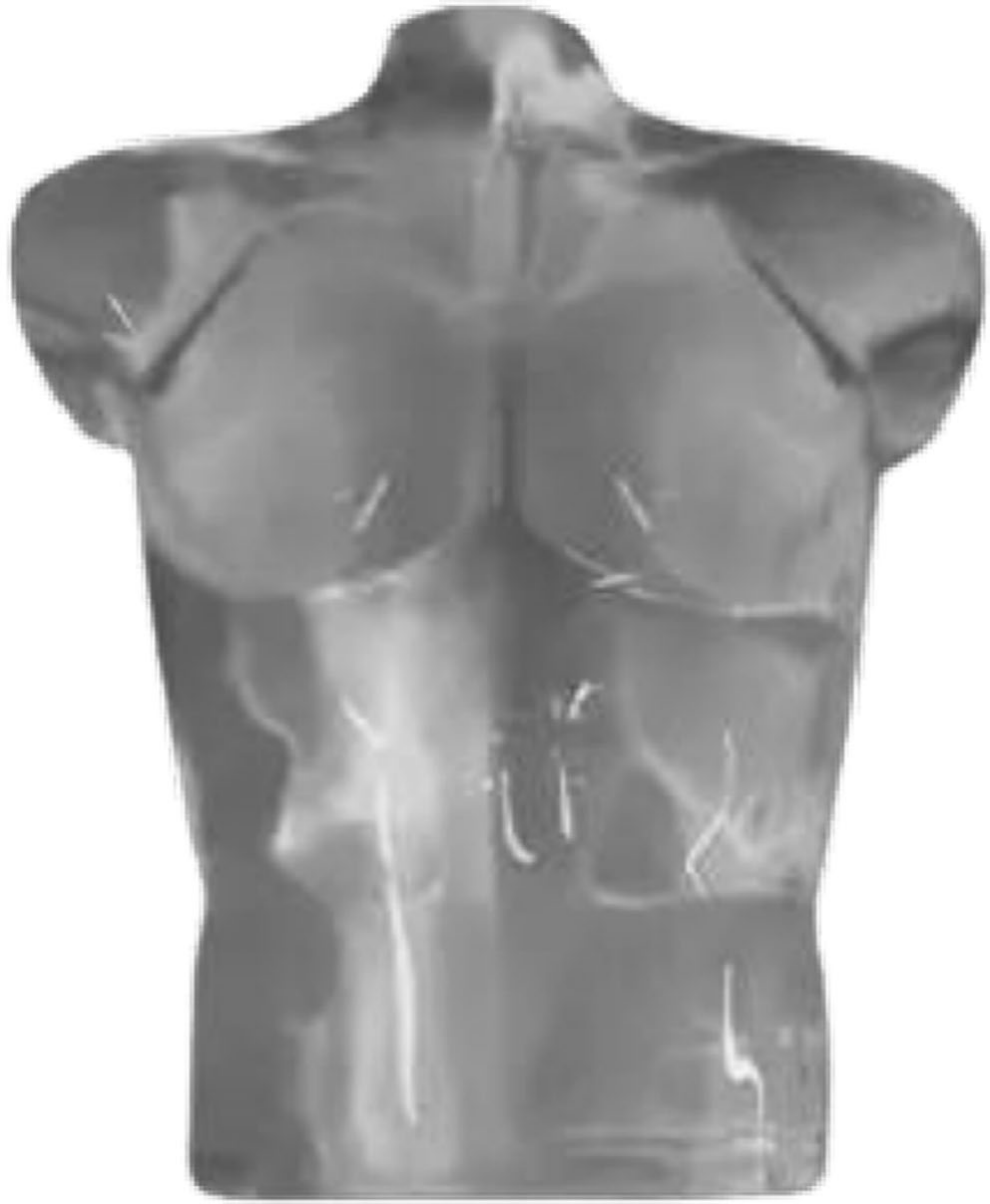
The torso phantom.

The second was a modified torso gel phantom. The US faculty made an incision in the lateral wall and inserted a water‐filled reusable water balloon to mimic a tissue plane visible on US (Figure 2). The original torso phantom was purchased from www.clearballistics.com (10% Gel Joe Fit Torso—Reusable Ballistic Testing Dummy).

**FIGURE 3:**
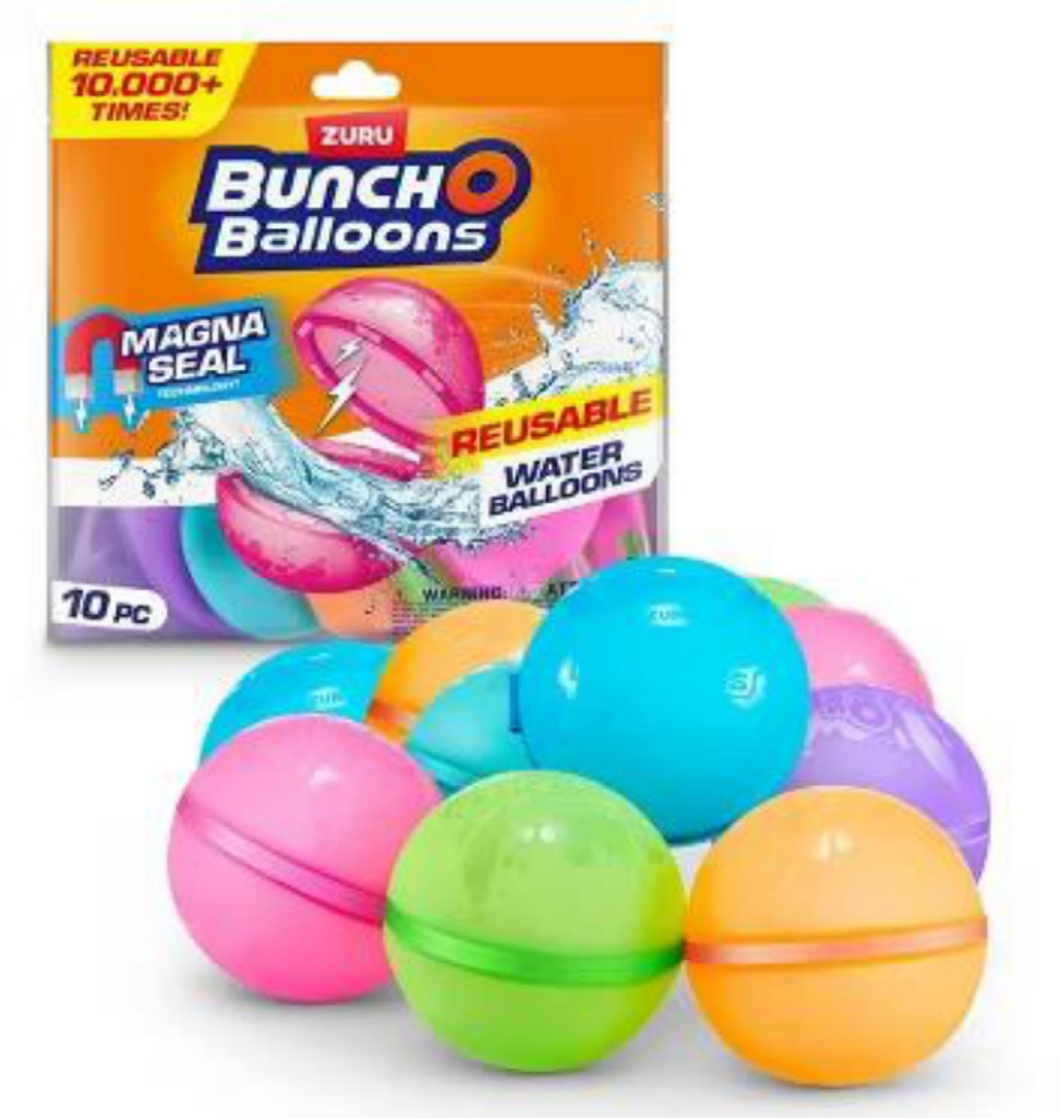
The reusable water balloons.

The reusable water balloons were from the brand, Zuru Toys (Zuru Group, El Segundo, CA) and can be found at major retailers (Figure 3).

Several blue block peripheral nerve gel phantoms were made available to learners to practice in‐plane US‐guided needle guidance. In addition, faculty created a custom serratus plane block phantom to use for needle technique practice. This phantom was created using a ballistic gel torso model which was modified via the insertion of a reusable water balloon to simulate a tissue plane which may be accessed for performance of this block.

### Post‐workshop assessments

**FIGURE 4:**
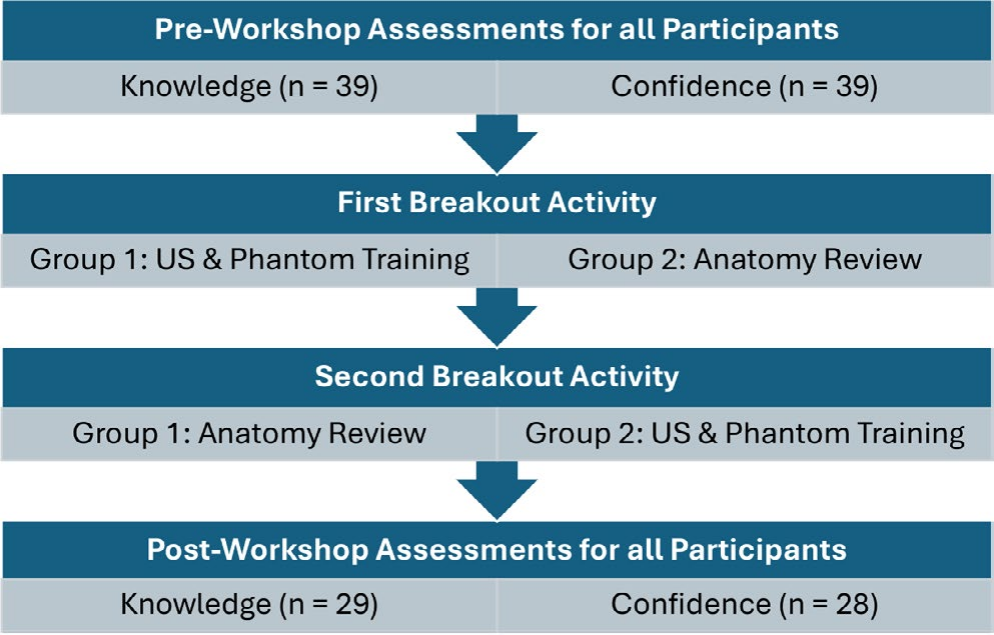
The flow of the workshop, including all activities and assessments.

After completing their respective activities, both groups switched. Immediately following the workshop activities, participants completed post‐workshop assessments. The post‐workshop knowledge assessment consisted of 16 multiple‐choice questions that were similar in nature, but not identical, to the pre‐workshop knowledge assessment. The post‐workshop confidence assessment had the same 16 Likert scale confidence questions asked in the pre‐workshop confidence assessment. The flow of the workshop is described in Figure 4. All assessments can be found in the Supporting Information.

### Data analysis

Data was assessed for normality using the Shapiro–Wilk test. Data from the pre‐ and post‐workshop confidence assessments' overall means were normally distributed and thus were compared using a paired *t*‐test. Data from the pre‐ and post‐workshop knowledge assessments and each individual question on the confidence assessments were not normally distributed, and thus the non‐parametric Wilcoxon signed‐rank test was used to compare. The Bonferroni correction was used when comparing confidence by individual question. Data from all PGYs in confidence and knowledge assessments were combined for overall analysis. Internal consistency was assessed using Cronbach's alpha.

## RESULTS

### Participant demographics

Participants ranged between first and fourth Post‐Graduate Year (PGY). The number of participants for each PGY is shown in Table 1.

**TABLE 1:** Shows participant completion of assessments by post‐graduate year (PGY).

Assessment	PGY 1	PGY 2	PGY 3	PGY 4	Unknown
Pre‐workshop confidence	13	14	11	2	–
Pre‐workshop knowledge	12	14	9	2	2
Post‐workshop confidence	11	8	6	2	1
Post‐workshop knowledge	10	9	6	2	2

### Anatomical knowledge

Forty‐four EM residents participated in the anatomy review and UGRA workshop (*n* = 44). Thirty‐nine (88.6%) participants completed the pre‐workshop knowledge assessment and 29 (65.9%) completed the post‐workshop knowledge assessment. Residents scored significantly higher on the post‐workshop knowledge assessment compared to the pre‐workshop knowledge assessment (12.0 ± 2.0 vs. 8.44 ± 2.45; *p* < 0.001) (Table 1). Table 2 compares mean knowledge scores by assessment period among participants.

**TABLE 2:** Comparison of knowledge assessments among all participants.

Knowledge assessment	*n*	Mean ± SD	High score	Low score
Pre‐workshop	39	8.44 ± 2.45	14	4
Post‐workshop	29	12.0 ± 2.0	15	8

*Note*: The post‐workshop mean knowledge score was significantly higher than the pre‐workshop mean (*p* < 0.001).

### Confidence results

Forty (90.9%) participants completed the pre‐workshop confidence assessment and 28 (63.6%) completed the post‐workshop confidence assessment. The responses received for “not at all confident” and “slightly confident” statements accounted for 69.3% of total responses in the pre‐workshop confidence assessment and decreased to 13.2% in the post‐workshop assessment. The responses received for “extremely confident” and “very confident” statements accounted for 11.1% of total responses in the pre‐workshop confidence assessment and increased to 45.1% in the post‐workshop assessment. Table 3 compares mean confidence scores between the assessments.

**TABLE 3:** Compares confidence mean and standard deviation by assessment period.

Assessment period	Mean ± SD
Pre‐workshop	2.06 ± 1.08
Post‐workshop	3.44 ± 0.89

*Note*: The post‐workshop confidence assessment was significantly higher when compared to the pre‐workshop confidence assessment (*p* < 0.001).

Table 4 shows the mean and standard deviation for the 16 confidence questions between the confidence assessments. Before the workshop, residents had the highest level of confidence in identifying the femoral nerve and artery (2.92 ± 1.46) and the lowest confidence in identifying the erector spinae muscle group (1.49 ± 0.68) on US imaging. At the end of the workshop, confidence in identifying the femoral nerve and artery (3.96 ± 0.88) remained the highest, while confidence in recognizing the difference between the trapezius and rhomboid muscles (2.96 ± 0.92) on US imaging was the lowest.

**TABLE 4:** Comparison of means and standard deviations by question between confidence assessments.

Confidence question	Confidence assessment	
	Pre‐workshop	Post‐workshop
How confident are you that you can simultaneously use an ultrasound probe and correct in‐plane needling technique during a nerve block procedure?	2.88 ± 1.11	3.61 ± 0.92
How confident are you that you can identify nerves on ultrasound imaging?	2.70 ± 0.91	3.39 ± 0.69
How confident are you that you can identify Erb's point and the posterior border of the sternocleidomastoid muscle on ultrasound imaging?	1.65 ± 0.92	3.29 ± 0.81
How confident are you that you can identify ribs and serratus anterior muscle along the midaxillary line on ultrasound imaging?	1.95 ± 1.01	3.43 ± 0.84
How confident are you that you can identify the fascial planes deep and superficial to the serratus anterior muscle on ultrasound imaging?	1.80 ± 0.88	3.29 ± 0.81
How confident are you that you can identify the thoracodorsal artery on ultrasound imaging?	1.65 ± 0.92	3.04 ± 1.00
How confident are you that you can identify the difference between the radial nerve and radial artery/veins on ultrasound imaging?	2.30 ± 1.14	3.82 ± 0.86
How confident are you that you can identify the difference between the ulnar nerve and ulnar artery/veins on ultrasound imaging?	2.30 ± 1.09	3.79 ± 0.88
How confident are you that you can identify the median nerve from other forearm structures on ultrasound imaging?	2.10 ± 1.10	3.71 ± 0.71
How confident are you that you can identify and differentiate the trapezius and rhomboid muscles on ultrasound imaging?	1.54 ± 0.68	2.96 ± 0.92
How confident are you that you can identify the erector spinae muscle group on ultrasound imaging?	1.49 ± 0.68	3.29 ± 0.94
How confident are you that you can identify transverse processes on ultrasound imaging?	2.15 ± 1.09	3.54 ± 0.79
How confident are you that you can identify the femoral head, anterior inferior iliac spine (AIIS) and iliopubic eminence on ultrasound imaging?	1.95 ± 0.94	3.21 ± 0.88
How confident are you that you can identify the femoral artery and femoral nerve on ultrasound imaging?	2.92 ± 1.46	3.96 ± 0.88
How confident are you that you can identify the subfascial plane deep to the psoas tendon on ultrasound imaging?	1.82 ± 0.85	3.14 ± 0.80
How confident are you that you can identify the tibial nerve among the other contents in the tarsal tunnel?	1.69 ± 0.77	3.50 ± 0.88
	*n* = 40	*n* = 28

**FIGURE 5:**
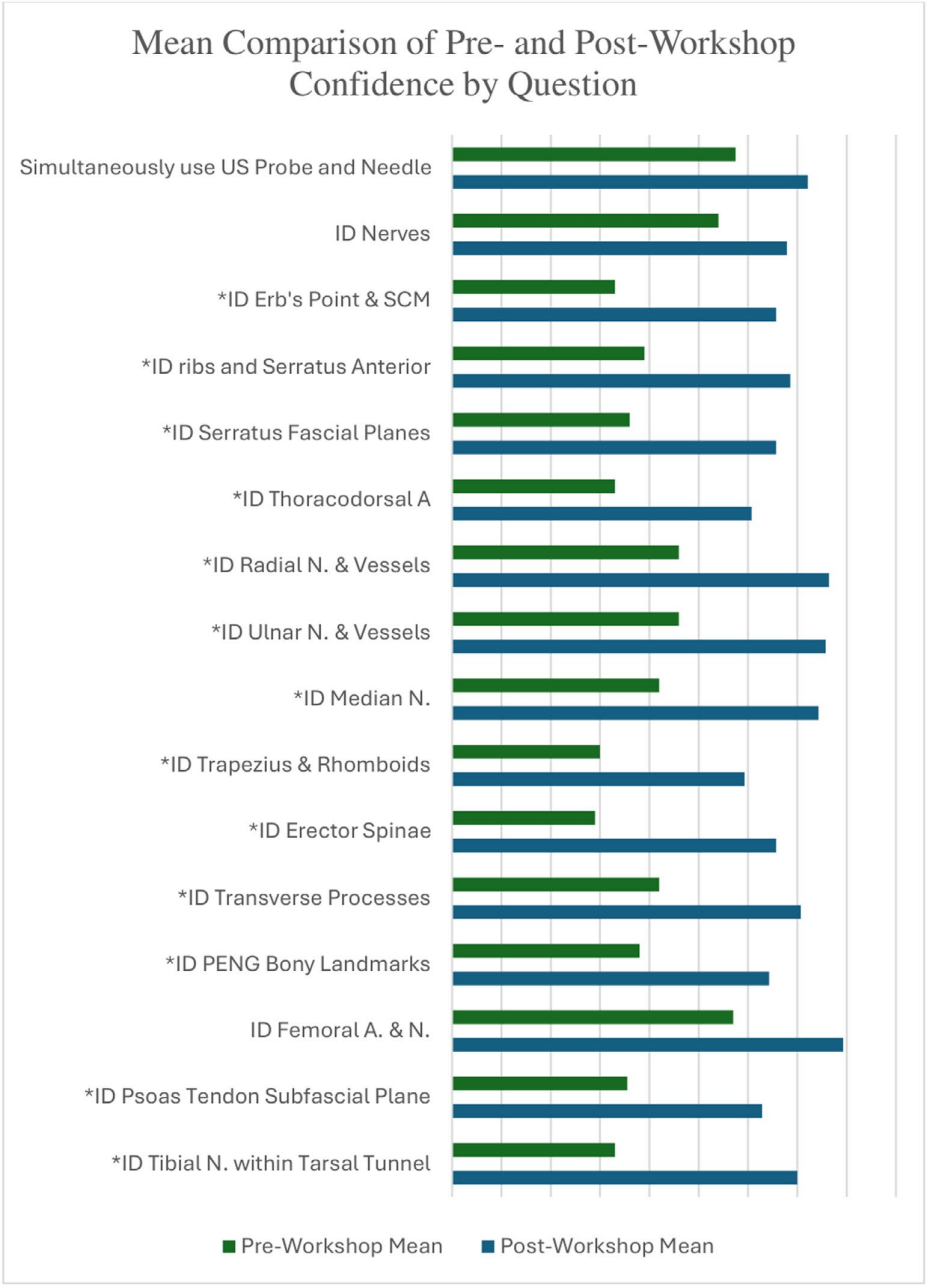
Comparison of mean confidence scores by question between the pre‐ and post‐workshop confidence assessments. When using the Bonferroni correction, 13 questions had a significant increase in mean scores (**p < 0.001).

A significant increase in residents' overall confidence was found on the post‐workshop assessment (*p* < 0.001). Thirteen questions individually received significantly higher confidence on the post‐workshop assessment when accounting for the Bonferroni correction (Figure 5). Cronbach's alpha coefficient was 0.939 for the confidence survey, suggesting the assessment measured with acceptable internal consistency.

## DISCUSSION

In this study, we assessed the implementation of a UGRA workshop utilizing body donor anatomical review, US scanning on standardized patients, and in‐plane needling technique on phantom models for EM residents. Using only a 50‐min anatomy review and a 50‐min session on US scanning and needle technique resulted in significant increases in resident knowledge and confidence. To the authors' knowledge, no other workshop focusing on these UGRA procedures has been described in the literature.

As pain continues to be one of the main causes of visits to the ED,^2^, ^18^ organizations such as the American College of Emergency Physicians have stated that UGRA should be a core component of pain control in the ED. The results of this study demonstrate that this workshop is a potential introductory option for teaching UGRA skills with which physicians can provide significant analgesia while also avoiding addictive medications such as opioids.

Results from the present study mirrored those of similar workshops. Tucker et al.^6^ also demonstrated an overwhelming increase in resident confidence after completing an UGRA curriculum. Similar methods were used between the two studies as both focused on anatomical landmarks using US and UGRA simulation. A similar study with EM residents found that their participants' knowledge of the interscalene plexus block improved significantly upon workshop completion.^11^ In a separate study, EM residents were overwhelmingly satisfied and stated they would attend an UGRA workshop again.^7^ The comparable results among these studies show how confidence can be increased through UGRA workshops.

Mean confidence scores for all questions were found to be significantly higher in the post‐workshop assessment and, in some cases, improvement was substantial. Inclusion of increased confidence for each statement demonstrates the workshop's effectiveness in achieving its goal for increasing participants' confidence in their abilities to successfully perform UGRA procedures. Our confidence results were not only similar to studies focusing on EM education^19^, ^20^ but also to workshops for anesthesiology residents.^12^, ^13^ Both studies^12^, ^13^ utilized prosected body donors for their respective workshops. Results from all studies indicate that reviewing anatomy on prosected body donors leads to greater confidence in residents' anatomical knowledge.

Similar to the results of an UGRA workshop for anesthesiology residents,^12^ we found our participants to have the highest confidence in recognizing the femoral nerve both before and after the workshop. While confidence in recognizing the tibial nerve in the tarsal tunnel was low in the present study, it was not the lowest rated as reported in the study by Harmon et al.^12^

The present study introduced innovation within UGRA education for EM residents by creating a workshop that eliminated traditional didactics while including an anatomical review on prosected donors and in‐plane needle‐to‐target practice on a phantom model. To the authors' knowledge, no workshop has been created using these same methods. While we closely followed methods for an anatomy review from a study by Harmon et al.,^12^ they did not include an opportunity for participants to practice in‐plane needle‐to‐target practice on a phantom model and their workshop did not include EM residents. Other workshops created for EM residents varied from the methods in this paper by either not including an anatomy review on prosected donors^6^, ^19^, ^20^ or not offering in‐plane needle‐to‐target practice on a phantom model but rather utilizing donor tissue.^19^, ^20^

The UGRA educational needs for EM residents are unique as many experienced faculty members may not be comfortable teaching these procedures as UGRA has been more recently adapted compared to traditional palpation‐based approaches. The need for proficient providers of these procedures within EM has been demonstrated by the American College of Emergency Physicians founding of a pain management section.^21^ To meet these needs, workshops such as the one described in this paper are needed to educate future EM physicians.

UGRA education is expanding within post‐graduate medical education, with many studies being conducted among various residency programs, including anesthesiology^12^, ^13^, ^14^, ^15^, ^22^, ^23^, ^24^ and emergency medicine.^6^, ^7^, ^11^, ^19^, ^20^, ^25^, ^26^ As educational methods slightly varied for some of these projects, future studies could incorporate multiple strategies to determine the most effective educational modalities.

A major goal of this workshop was to present material in an active manner to motivate participants and encourage short‐term knowledge growth. Active learning increases learners' attention and engagement, leading to longer retention of knowledge.^27^ Active learning has demonstrated the ability to extend knowledge gains beyond the short term by inviting learners to participate throughout educational interventions. A benefit of short‐term sustainability is immediate realization in achieving objectives and goals.^28^ As this workshop focused on short‐term knowledge growth, participants were able to immediately realize newly acquired knowledge. The sustainability of short‐term knowledge gain was demonstrated in a professional development (PD) workshop for educators. Well after completing the PD workshop, participants displayed knowledge retention and positive influence gained from the intervention, 15 months later.^29^

In the future, our study will be expanded to include different cohorts to improve the reliability of this workshop. EM residents from another institution and medical students at OSU will be invited to participate in the workshop. This workshop will also be implemented into the EM residency curriculum at OSU. A further study will be created by EM faculty members to track the number of UGRA procedures performed in the ED to determine the effectiveness of this workshop and the improvement of participants' performance for UGRA procedures. Most importantly, future studies will include assessment of clinical competency following training.

## LIMITATIONS

Limitations of this study include working with a single field of medicine (EM) at one institution (OSU) in a single year, leading to a relatively low number of participants (*n* = 44). Furthermore, the dropout of participants throughout the study is a major limitation. While EM residency leadership required residents to be present for the workshop, they were not required to participate in the study. Furthermore, some participants had recently completed an overnight shift and arrived late, leading to incompletions of pre‐workshop assessments. Other residents had to leave early for clinical duties leading to incompletions of post‐workshop assessments. It is difficult to demonstrate the effectiveness of the anatomical review (whether on donors or with US scanning) without participants completing the post‐workshop assessments. This is a potential explanation for not all participants completing every assessment. The assessments themselves are another potential limitation, as their validity and reliability have not been tested in similar workshops, though measures of internal consistency and difficulty were reassuring.

A limitation of the body of literature on EM education regarding UGRA is the lack of higher‐level Kirkpatrick outcomes. Kirkpatrick outcomes evaluate educational/training programs with higher‐level outcomes centered on learners' transfer of knowledge and influences the training had on them. While we did not show changes in behavior following this single workshop, we believe the rigor of our confidence and knowledge assessments adds to the support for these teaching techniques. Following up with a broader and larger learner population as well as assessment of competency or behavior outcomes, would certainly be exciting areas for future study.

Another limitation of this study is the lack of measurement of participants' performance of UGRA procedures. At the time of the study, very few UGRA procedures were being performed within the EM department (including faculty members), making it difficult to measure practical performance for the participants. Future studies will analyze residents' performance in simulated scenarios once they are exposed to more repetition of these procedures.

## CONCLUSIONS

As the need for alternative methods for pain control in the ED continues to be sought after, UGRA instruction is critical to achieve this within graduate medical education. The self‐efficacy gained by residents in this workshop can lead to increased patient safety as opioids are avoided in the ED. Workshops such as the one described in this manuscript not only provide residents an avenue to build their skills with these procedures, but also provide attendings who did not have the opportunity to learn these skills. Settings such as this workshop provide EM faculty members an effective environment in which to teach their residents proper skills for UGRA and can be easily replicated at other institutions.

## AUTHOR CONTRIBUTIONS

**Geoffery D. Fernquist:** Conceptualization; methodology; formal analysis; resources; writing – original draft. **Derek Harmon:** Writing – review and editing; supervision. **Lauren D. Branditz:** Conceptualization; methodology. **Andrew Kendle:** Conceptualization; methodology. **Melissa M. Quinn:** Writing – review and editing; supervision.

## FUNDING INFORMATION

This study was supported by the Division of Anatomy, Department of Biomedical Education and Anatomy, The Ohio State University College of Medicine, Columbus, Ohio, USA.

## CONFLICT OF INTEREST STATEMENT

The authors report no conflict of interest.

## ETHICS STATEMENT

All participants agreed to the terms of the workshop and none were coerced into participating. No Protected Health Information was accessed nor used for this study.

## Supporting information


Data S1.

Data S2.

## Data Availability

The data used in this study have not and are not being used in any other manuscript.
