# Supplementary material for: A novel nerve block and anatomy workshop for emergency medicine residents: A pilot study
Source: Anat Sci Educ. 2026 Apr 14;19(7):1191–200. doi: 10.1002/ase.70240 (PMC13332513; doi:10.1002/ase.70240)
Supplement: Supplementary file 1 — Data S1. [file ASE-19-1191-s002.docx]

Pre-Confidence Assessment

I.

| 1. What is your name? |  | |
| --- | --- | --- |
| **OR** | | |
| 1. What is the name of the street you lived on growing up?   (For purposes of linking the assessments together) |  | |
| 1. What was the color of your first car?   (For purposes of linking the assessments together) |  | |
|  | | |
| 1. Did you participate in this workshop last year? | Yes No | |
| 1. What is your current PGY? |  | |
| 1. How many years have passed since your last formal anatomy course? | ­­­________ | |
| 1. Were cadavers used in the anatomy course? | ________ | |
| 1. Did you dissect in the anatomy course? | ________ | |
| 1. Was your anatomy course virtual or in person? | Virtual | In-person |
| 1. How many times have you previously observed a nerve block procedure? | ________ | |
| 1. How many times have you performed a nerve block procedure (or assisted)? | ________ | |
| 1. What is the name of the street you lived on growing up?   (For purposes of linking the assessments together) |  | |
| 1. What was the color of your first car?   (For purposes of linking the assessments together) |  | |

II. **Please respond to the following questions regarding your confidence. Circle one answer for each statement.**

| **How confident are you that you can…** | **Choose the option that most closely aligns with your confidence** | | | | |
| --- | --- | --- | --- | --- | --- |
| 1. Simultaneously use an ultrasound probe and correct in-plane needling technique during a nerve block procedure | Extremely Confident | Very Confident | Moderately Confident | Slightly Confident | Not at all Confident |
| 1. Identify nerves on ultrasound imaging | Extremely Confident | Very Confident | Moderately Confident | Slightly Confident | Not at all Confident |
| 1. Regarding the **superficial cervical plexus block** rate your confidence in your ability to:    1. Identify Erb’s Point (nerve point of the neck) and the posterior border of the sternocleidomastoid muscle on ultrasound imaging | Extremely Confident | Very Confident | Moderately Confident | Slightly Confident | Not at all Confident |
| 1. Regarding the **serratus anterior plane block** rate your confidence in your ability to:    1. Identify ribs and serratus anterior muscle along the midaxillary line on ultrasound imaging    2. Identify the fascial planes deep and superficial to the serratus anterior muscle on ultrasound imaging    3. Identify the thoracodorsal artery on ultrasound imaging | Extremely Confident  Extremely Confident  Extremely Confident | Very Confident  Very Confident  Very Confident | Moderately Confident  Moderately Confident  Moderately Confident | Slightly Confident  Slightly Confident  Slightly Confident | Not at all Confident  Not at all Confident  Not at all Confident |
| 1. Regarding the **radial nerve block** rate your confidence in your ability to:    1. Identify the difference between the radial nerve and radial artery/veins on ultrasound imaging | Extremely Confident | Very Confident | Moderately Confident | Slightly Confident | Not at all Confident |
| 1. Regarding the **ulnar nerve block** rate your confidence in your ability to:    1. Identify the difference between the ulnar nerve and ulnar artery/veins on ultrasound imaging | Extremely Confident | Very Confident | Moderately Confident | Slightly Confident | Not at all Confident |
| 1. Regarding the **median nerve block** rate your confidence in your ability to:    1. Identify the median nerve from other forearm structures on ultrasound imaging | Extremely Confident | Very Confident | Moderately Confident | Slightly Confident | Not at all Confident |
| 1. Regarding the **erector spinae plane block** rate your confidence in your ability to:    1. Identify and differentiate the trapezius and rhomboid muscles on ultrasound imaging    2. Identify the erector spinae muscle group on ultrasound imaging    3. Identify transverse processes on ultrasound imaging | Extremely Confident  Extremely Confident  Extremely Confident | Very Confident  Very Confident  Very Confident | Moderately Confident  Moderately Confident  Moderately Confident | Slightly Confident  Slightly Confident  Slightly Confident | Not at all Confident  Not at all Confident  Not at all Confident |
| 1. Regarding the **PENG block** rate your confidence in your ability to:    1. Identify the femoral head, anterior inferior iliac spine (AIIS) and iliopubic eminence on ultrasound imaging    2. Identify the femoral artery and femoral nerve on ultrasound imaging    3. Identify the subfascial plane deep to the psoas tendon on ultrasound imaging | Extremely Confident  Extremely Confident  Extremely Confident | Very Confident  Very Confident  Very Confident | Moderately Confident  Moderately Confident  Moderately Confident | Slightly Confident  Slightly Confident  Slightly Confident | Not at all Confident  Not at all Confident  Not at all Confident |
| 1. Regarding the **tibial nerve block** rate your confidence in your ability to:    1. Identify the tibial nerve among the other contents in the tarsal tunnel | Extremely Confident | Very Confident | Moderately Confident | Slightly Confident | Not at all Confident |

Post-Confidence Assessment

| What is the name of the street you lived on growing up?  (For purposes of linking the assessments together) | | |  | | | |
| --- | --- | --- | --- | --- | --- | --- |
| What was the color of your first car?  (For purposes of linking the assessments together) | | |  | | | |
| **OR** | | | | | | |
| What is your name | | |  | | | |
| **Please respond to the following questions regarding your confidence. Circle one answer for each statement.** | | | | | | |
| **How confident are you that you can…** | **Choose the option that most closely aligns with your confidence** | | | | | |
| 1. Simultaneously use an ultrasound probe and correct in-plane needling technique during a nerve block procedure | Extremely Confident | Very Confident | | Moderately Confident | Slightly Confident | Not at all Confident |
| 1. Identify nerves on ultrasound imaging | Extremely Confident | Very Confident | | Moderately Confident | Slightly Confident | Not at all Confident |
| 1. Regarding the **superficial cervical plexus block** rate your confidence in your ability to:    1. Identify Erb’s Point (nerve point of the neck) and the posterior border of the sternocleidomastoid muscle on ultrasound imaging | Extremely Confident | Very Confident | | Moderately Confident | Slightly Confident | Not at all Confident |
| 1. Regarding the **serratus anterior plane block** rate your confidence in your ability to:    1. Identify ribs and serratus anterior muscle along the midaxillary line on ultrasound imaging    2. Identify the fascial planes deep and superficial to the serratus anterior muscle on ultrasound imaging    3. Identify the thoracodorsal artery on ultrasound imaging | Extremely Confident  Extremely Confident  Extremely Confident | Very Confident  Very Confident  Very Confident | | Moderately Confident  Moderately Confident  Moderately Confident | Slightly Confident  Slightly Confident  Slightly Confident | Not at all Confident  Not at all Confident  Not at all Confident |
| 1. Regarding the **radial nerve block** rate your confidence in your ability to:    1. Identify the difference between the radial nerve and radial artery/veins on ultrasound imaging | Extremely Confident | Very Confident | | Moderately Confident | Slightly Confident | Not at all Confident |
| 1. Regarding the **ulnar nerve block** rate your confidence in your ability to:    1. Identify the difference between the ulnar nerve and ulnar artery/veins on ultrasound imaging | Extremely Confident | Very Confident | | Moderately Confident | Slightly Confident | Not at all Confident |
| 1. Regarding the **median nerve block** rate your confidence in your ability to:    1. Identify the median nerve from other forearm structures on ultrasound imaging | Extremely Confident | Very Confident | | Moderately Confident | Slightly Confident | Not at all Confident |
| 1. Regarding the **erector spinae plane block** rate your confidence in your ability to:    1. Identify and differentiate the trapezius and rhomboid muscles on ultrasound imaging    2. Identify the erector spinae muscle group on ultrasound imaging    3. Identify transverse processes on ultrasound imaging | Extremely Confident  Extremely Confident  Extremely Confident | Very Confident  Very Confident  Very Confident | | Moderately Confident  Moderately Confident  Moderately Confident | Slightly Confident  Slightly Confident  Slightly Confident | Not at all Confident  Not at all Confident  Not at all Confident |
| 1. Regarding the **PENG block** rate your confidence in your ability to:    1. Identify the femoral head, anterior inferior iliac spine (AIIS) and iliopubic eminence on ultrasound imaging    2. Identify the femoral artery and femoral nerve on ultrasound imaging    3. Identify the subfascial plane deep to the psoas tendon on ultrasound imaging | Extremely Confident  Extremely Confident  Extremely Confident | Very Confident  Very Confident  Very Confident | | Moderately Confident  Moderately Confident  Moderately Confident | Slightly Confident  Slightly Confident  Slightly Confident | Not at all Confident  Not at all Confident  Not at all Confident |
| 1. Regarding the **tibial nerve block** rate your confidence in your ability to:    1. Identify the tibial nerve among the other contents in the tarsal tunnel | Extremely Confident | Very Confident | | Moderately Confident | Slightly Confident | Not at all Confident |

Three Month Follow-up Confidence Assessment

Please respond to the following questions regarding your confidence. Circle one answer for each statement.

| What is the name of the street you lived on growing up?  (For purposes of linking the assessments together) | | |  | | | | | |
| --- | --- | --- | --- | --- | --- | --- | --- | --- |
| What was the color of your first car?  (For purposes of linking the assessments together) | | |  | | | | | |
| **OR** | | | | | | | | |
| What is your name | | |  | | | | | |
|  | | | | | | | | |
| **How confident are you that you can…** | **Choose the option that most closely aligns with your confidence** | | | | | | | |
| 1. Simultaneously use an ultrasound probe and correct in-plane needling technique during a nerve block procedure | Extremely Confident | Very Confident | | | Moderately Confident | Slightly Confident | | Not at all Confident |
| 1. Identify nerves on ultrasound imaging | Extremely Confident | Very Confident | | | Moderately Confident | Slightly Confident | | Not at all Confident |
| 1. Regarding the **superficial cervical plexus block** rate your confidence in your ability to:    1. Identify Erb’s Point (nerve point of the neck) and the posterior border of the sternocleidomastoid muscle on ultrasound imaging | Extremely Confident | Very Confident | | | Moderately Confident | Slightly Confident | | Not at all Confident |
| 1. Regarding the **serratus anterior plane block** rate your confidence in your ability to:    1. Identify ribs and serratus anterior muscle along the midaxillary line on ultrasound imaging    2. Identify the fascial planes deep and superficial to the serratus anterior muscle on ultrasound imaging    3. Identify the thoracodorsal artery on ultrasound imaging | Extremely Confident  Extremely Confident  Extremely Confident | Very Confident  Very Confident  Very Confident | | | Moderately Confident  Moderately Confident  Moderately Confident | Slightly Confident  Slightly Confident  Slightly Confident | | Not at all Confident  Not at all Confident  Not at all Confident |
| 1. Regarding the **radial nerve block** rate your confidence in your ability to:    1. Identify the difference between the radial nerve and radial artery/veins on ultrasound imaging | Extremely Confident | Very Confident | | | Moderately Confident | Slightly Confident | | Not at all Confident |
| 1. Regarding the **ulnar nerve block** rate your confidence in your ability to:    1. Identify the difference between the ulnar nerve and ulnar artery/veins on ultrasound imaging | Extremely Confident | Very Confident | | | Moderately Confident | Slightly Confident | | Not at all Confident |
| 1. Regarding the **median nerve block** rate your confidence in your ability to:    1. Identify the median nerve from other forearm structures on ultrasound imaging | Extremely Confident | Very Confident | | | Moderately Confident | Slightly Confident | | Not at all Confident |
| 1. Regarding the **erector spinae plane block** rate your confidence in your ability to:    1. Identify and differentiate the trapezius and rhomboid muscles on ultrasound imaging    2. Identify the erector spinae muscle group on ultrasound imaging    3. Identify transverse processes on ultrasound imaging | Extremely Confident  Extremely Confident  Extremely Confident | Very Confident  Very Confident  Very Confident | | | Moderately Confident  Moderately Confident  Moderately Confident | Slightly Confident  Slightly Confident  Slightly Confident | | Not at all Confident  Not at all Confident  Not at all Confident |
| 1. Regarding the **PENG block** rate your confidence in your ability to:    1. Identify the femoral head, anterior inferior iliac spine (AIIS) and iliopubic eminence on ultrasound imaging    2. Identify the femoral artery and femoral nerve on ultrasound imaging    3. Identify the subfascial plane deep to the psoas tendon on ultrasound imaging | Extremely Confident  Extremely Confident  Extremely Confident | Very Confident  Very Confident  Very Confident | | | Moderately Confident  Moderately Confident  Moderately Confident | Slightly Confident  Slightly Confident  Slightly Confident | | Not at all Confident  Not at all Confident  Not at all Confident |
| 1. Regarding the **tibial nerve block** rate your confidence in your ability to:    1. Identify the tibial nerve among the other contents in the tarsal tunnel | Extremely Confident | Very Confident | | | Moderately Confident | Slightly Confident | | Not at all Confident |
| 1. How many times have you previously observed a nerve block procedure? | | | | ________ | | | | |
| 1. How many times have you performed a nerve block procedure (or assisted)? | | | | ________ | | | | |
| 1. Can the knowledge you gained from the workshop be applied to procedures other than nerve blocks? | | | | Yes | | | No | |
| 1. Would you recommend this workshop to other residents? | | | | Yes | | | No | |
| 1. Rate the importance of the workshops’ cadaveric anatomy review for your ability to administer nerve blocks | Extremely Important | Very Important | | | Moderately Important | Slightly Important | | Not at all Important |
| 1. Rate the importance of the workshops’ ultrasound scanning practice for your ability to administer nerve blocks | Extremely Important | Very Important | | | Moderately Important | Slightly Important | | Not at all Important |
