# Supplementary material for: A novel nerve block and anatomy workshop for emergency medicine residents: A pilot study
Source: Anat Sci Educ. 2026 Apr 14;19(7):1191–200. doi: 10.1002/ase.70240 (PMC13332513; doi:10.1002/ase.70240)
Supplement: Supplementary file 2 — Data S2. [file ASE-19-1191-s001.docx]

Pre-Assessment

Name_________________________

OR

What is the name of the street you lived on growing up? (For purposes of linking the assessments together) ___________________

What was the color of your first car? (For purposes of linking the assessments together)

_____________________

1. A patient enters the ER with a clavicular fracture, what nerve block is the most appropriate to alleviate pain?
   1. **Superficial cervical plexus block**
   2. Axillary block
   3. Infraclavicular block
   4. Supraclavicular block
2. Which muscle sits directly anterior to the nerves of the superficial cervical plexus (Erb’s Point)?
   1. Thyrohyoid
   2. Sternothyroid
   3. **Sternocleidomastoid**
   4. Omohyoid Superior Belly
3. Which of the following nerves can be affected by a serratus plane block?
   1. **Intercostal nerves, Thoracodorsal nerve, Long Thoracic nerve**
   2. Lower Subscapular nerve, Intercostal nerves, Dorsal Scapular nerve
   3. Dorsal scapular nerve, Intercostal nerves, Long Thoracic nerve
   4. Long thoracic nerve, Thoracodorsal nerve, Lower Subscapular nerve
4. What artery must be avoided during a serratus plane block?
   1. Dorsal scapular a.
   2. Circumflex scapular a.
   3. **Thoracodorsal a.**
   4. Subscapular a
5. A patient has multiple lacerations on the lateral, palmar aspect of the hand not involving the thumb. What nerve requires anesthesia?
   1. Ulnar
   2. Radial
   3. **Median**
   4. Musculocutaneous
6. While scanning the middle of the anterior forearm, you see the image below. What nerve is the arrow pointing to?


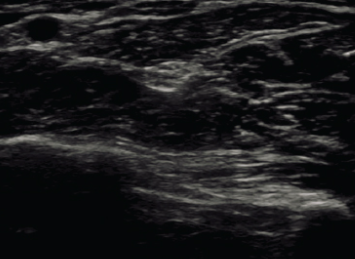


(Figure 4.27)

- 1. Ulnar
  2. **Median**
  3. Radial
  4. Musculocutaneous

1. The green area (indicated by the arrow) in the image below shows the cutaneous innervation for which nerve?


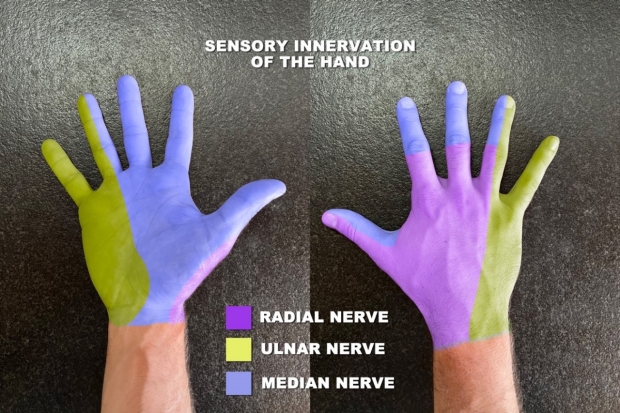


- 1. **Ulnar n.**
  2. Median n.
  3. Radial n.
  4. Musculocutaneous n.

1. A block was performed on a patient’s neck to alleviate pain in their upper extremity, but they are still experiencing pain in the most medial part of their hand and wrist. What additional block will the patient require?
   1. Median n.
   2. **Ulnar n.**
   3. Radial n.
   4. Musculocutaneous n.
2. Where would you administer a radial nerve block to alleviate pain in the hand due to laceration?
   1. **Lateral aspect of wrist or proximal forearm**
   2. Posterior aspect of the humeral medial epicondyle
   3. Medial, anterior aspect of the wrist
   4. Posterior midline of the wrist
3. You are performing a radial nerve block at the lateral elbow. What two muscles surround the radial nerve here?
   1. Biceps Brachii and Brachialis muscles
   2. Biceps Brachii and Brachioradialis muscles
   3. **Brachialis and Brachioradialis muscles**
   4. Brachialis and Coracobrachialis muscles
4. Which nerve’s articular branches are targets while using the PENG (pericapsular nerve group) block?
   1. Femoral, Sciatic and Obturator
   2. Sciatic, Obturator and Accessory Obturator
   3. **Femoral, Obturator and Accessory Obturator**
   4. Inferior Gluteal, Femoral and Sciatic
5. Which of the following options list two anatomical landmarks for the PENG block (pericapsular nerve group block)?
   1. Anterior Superior Iliac Spine and Greater Trochanter
   2. Anterior Inferior Iliac Spine and Greater Trochanter
   3. **Iliopubic eminence and Psoas Tendon**
   4. Psoas tendon and Anterior Superior Iliac Spine
6. A patient arrives with a fractured calcaneus. What nerve needs to be targeted to alleviate the pain?
   1. **Tibial n.**
   2. Sural n.
   3. Common fibular n.
   4. Lateral plantar n.
7. Which number corresponds with an area of the foot innervated by a branch of tibial nerve?


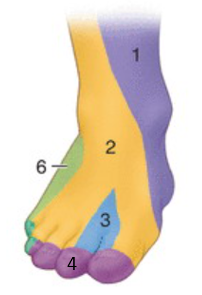
(Figure 7.74, Moore 2017)

- 1. 1
  2. 2
  3. 3
  4. **4**

1. When performing an erector spinae plane block, what is the targeted ‘backstop’ to prevent damage to surrounding critical structures?
   1. Vertebral laminae
   2. Spinous process
   3. **Transverse process**
   4. Rib angle
2. In the erector spinae muscle group, what muscle is most lateral?
   1. Longissimus
   2. Multifidus
   3. **Iliocostalis**
   4. Spinalis

Post Assessment

Name_________________________

OR

What is the name of the street you lived on growing up? (For purposes of linking the assessments together) ___________________

What was the color of your first car? (For purposes of linking the assessments together)

_____________________

1. A patient has a laceration to their ear lobe. Which of the following nerve blocks will provide pain relief?
   1. Supraclavicular block
   2. **Superficial cervical plexus block**
   3. Infraclavicular block
   4. Interscalene block
2. Which muscle sits directly anterior to the nerves of the superficial cervical plexus (Erb’s Point)?
   1. Thyrohyoid
   2. Sternothyroid
   3. **Sternocleidomastoid**
   4. Omohyoid Superior Belly
3. Other than the serratus anterior m., what muscle can experience paralysis during a serratus plane block?
   1. Rhomboid Major m.
   2. Levator Scapulae
   3. **Latissimus Dorsi m.**
   4. Teres Minor m.
4. What artery has the risk of being punctured during a serratus plane block?
   1. Dorsal scapular a.
   2. Circumflex scapular a.
   3. **Thoracodorsal a.**
   4. Subscapular a
5. A patient has a deep laceration on the medial-most palmar aspect of the hand (just proximal to the 5^th^ digit). What nerve requires anesthesia?
   1. **Ulnar**
   2. Radial
   3. Median
   4. Musculocutaneous
6. A patient fractured their humeral medial epicondyle. What nerve is most likely damaged?
   1. Median
   2. **Ulnar**
   3. Musculocutaneous
   4. Radial
7. The blue area (indicated by the arrow) in the image below shows the cutaneous innervation for which nerve?


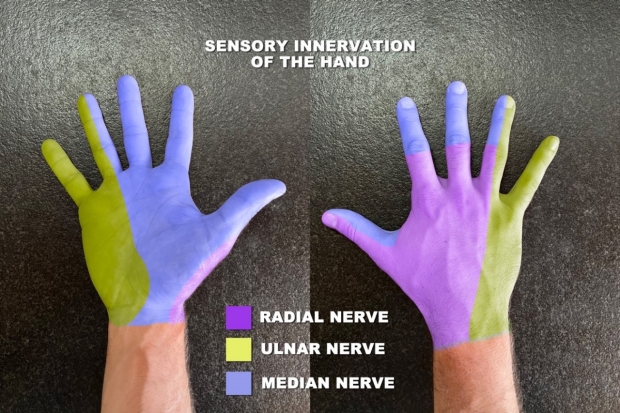


- 1. Ulnar n.
  2. **Median n.**
  3. Radial n.
  4. Musculocutaneous n.

1. What structure sits directly lateral to the median nerve within the cubital fossa?
   1. **Brachial artery**
   2. Biceps Brachii tendon
   3. Brachialis tendon
   4. Pronator Teres muscle
2. You are performing a radial nerve block at the lateral elbow. What two muscles surround the radial nerve here?
   1. Biceps Brachii and Brachialis muscles
   2. Biceps Brachii and Brachioradialis muscles
   3. **Brachialis and Brachioradialis muscles**
   4. Brachialis and Coracobrachialis muscles
3. What nerve has a major cutaneous branch that travels over the anatomical snuffbox?
   1. Median
   2. Ulnar
   3. **Radial**
   4. Musculocutaneous
4. Which of the following options list two anatomical landmarks for the PENG block (pericapsular nerve group block)?
   1. Anterior Superior Iliac Spine and Greater Trochanter
   2. Anterior Inferior Iliac Spine and Greater Trochanter
   3. **Iliopubic eminence and Psoas Tendon**
   4. Psoas tendon and Anterior Superior Iliac Spine
5. The PENG (pericapsular nerve group) block must be administered in the subfascial plane between what pair of structures?
   1. **Psoas tendon and ilium**
   2. Pectineus and pubis
   3. Iliacus and Iliopubic eminence
   4. Pectineus and iliopubic eminence
6. Which area would be affected by a tibial nerve block administered at the ankle?
   1. **Medial plantar aspect of the foot**
   2. Skin overlying the medial malleolus
   3. Skin overlying the lateral malleolus
   4. Mid-dorsum of the foot
7. Within the tarsal tunnel, the tibial nerve is directly anterior to which structure?
   1. Tibialis Posterior m.
   2. **Flexor Hallucis Longus m.**
   3. Flexor Digitorum Longus m.
   4. Posterior Tibial a/v.
8. For upper thoracic erector spinae blocks, besides the erector spinae muscles, which muscles must the needle advance through to properly administer the anesthesia?
   1. **Rhomboid major/minor & Trapezius**
   2. Teres major/minor & Trapezius
   3. Trapezius & Supraspinatus
   4. Trapezius & Infraspinatus
9. What is the innervation for the erector spinae muscles?
   1. Intercostal nerves
   2. Ventral Rami
   3. **Dorsal Rami**
   4. Lumbar and sacral plexuses

3 Month Follow Up

Name_________________________

OR

What is the name of the street you lived on growing up? (For purposes of linking the assessments together) ___________________

What was the color of your first car? (For purposes of linking the assessments together)

_____________________

1. Which nerve is found in Erb’s Point?
   1. Suboccipital n.
   2. **Supraclavicular n.**
   3. Third Occipital n.
   4. Posterior Auricular n.
2. **
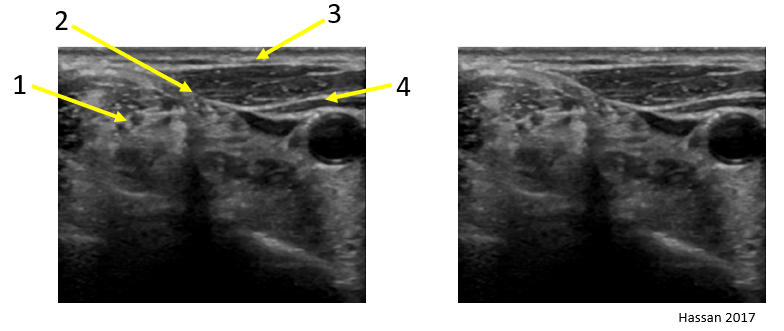
**Which arrow is pointing to the correct location for the superficial cervical plexus block?
   1. 1
   2. **2**
   3. 3
   4. 4
3. Which of the following nerves can be affected by a serratus plane block?
   1. **Intercostal nerves, Thoracodorsal nerve, Long Thoracic nerve**
   2. Lower Subscapular nerve, Intercostal nerves, Dorsal Scapular nerve
   3. Dorsal scapular nerve, Intercostal nerves, Long Thoracic nerve
   4. Long thoracic nerve, Thoracodorsal nerve, Lower Subscapular nerve
4. Other than the serratus anterior m., what muscle can experience paralysis during a serratus plane block?
   1. Rhomboid Major m.
   2. Levator Scapulae
   3. **Latissimus Dorsi m.**
   4. Teres Minor m.
5. A block was performed on a patient’s neck to alleviate pain in their upper extremity but they are still experiencing pain in the most medial part of their hand and wrist. What additional block will the patient require?
   1. Median n.
   2. **Ulnar n.**
   3. Radial n.
   4. Musculocutaneous n.
6. A patient fractured their humeral medial epicondyle. What nerve is most likely damaged?
   1. Median
   2. **Ulnar**
   3. Musculocutaneous
   4. Radial
7. A patient has multiple lacerations on the lateral, palmar aspect of the hand not involving the thumb. What nerve requires anesthesia?
   1. Ulnar
   2. Radial
   3. **Median**
   4. Musculocutaneous
8. What structure sits directly lateral to the median nerve within the cubital fossa?
   1. **Brachial artery**
   2. Biceps Brachii tendon
   3. Brachialis tendon
   4. Pronator Teres muscle
9. The purple area (indicated by the arow) in the image below shows the cutaneous innervation for which nerve?


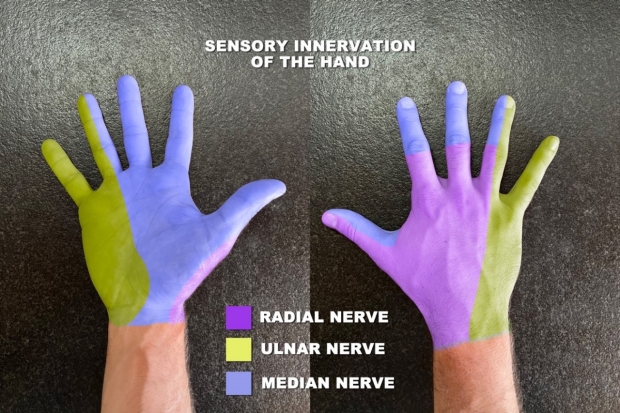


- 1. Ulnar n.
  2. Median n.
  3. **Radial n.**
  4. Musculocutaneous n.

1. Where would you administer a radial nerve block to alleviate pain in the hand due to laceration?
   1. **Lateral aspect of wrist or proximal forearm**
   2. Posterior aspect of the humeral medial epicondyle
   3. Medial, anterior aspect of the wrist
   4. Posterior midline of the wrist
2. Which nerve’s articular branches are targets while using the PENG (pericapsular nerve group) block?
   1. Femoral, Sciatic and Obturator
   2. Sciatic, Obturator and Accessory Obturator
   3. **Femoral, Obturator and Accessory Obturator**
   4. Inferior Gluteal, Femoral and Sciatic
3. The PENG (pericapsular nerve group) block must be administered in the subfascial plane between what pair of structures?
   1. **Psoas tendon and ilium**
   2. Pectineus and pubis
   3. Iliacus and Iliopubic eminence
   4. Pectineus and iliopubic eminence
4. Within the tarsal tunnel, which structure sits directly posterior to the tibial nerve?
   1. Tibialis Posterior m.
   2. **Flexor Hallucis Longus m.**
   3. Flexor Digitorum Longus m.
   4. Posterior Tibial a/v.
5. Which of the following options correctly lists the areas of the foot innervated by the tibial nerve on this image?


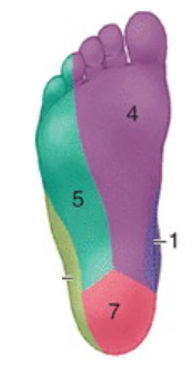
 (Figure 7.74, Moore 2017)

- 1. **Areas 4, 5 & 7**
  2. Areas 1, 4 & 5
  3. Areas 1, 5 & 7
  4. Areas 4, 1 & 7

1. In the erector spinae muscle group, what muscle is intermediate?
   1. **Longissimus**
   2. Multifidus
   3. Iliocostalis
   4. Spinalis
2. When performing an erector spinae plane block, what is the targeted ‘backstop’ to prevent damage to surrounding critical structures?
   1. Vertebral laminae
   2. Spinous process
   3. **Transverse process**
   4. Rib angle
